# Follow-Up of Cancer Patients Receiving Anti-PD-(L)1 Therapy Using an Electronic Patient-Reported Outcomes Tool (KISS): Prospective Feasibility Cohort Study

**DOI:** 10.2196/17898

**Published:** 2020-10-28

**Authors:** Sanna Iivanainen, Tuomo Alanko, Pia Vihinen, Teemu Konkola, Jussi Ekstrom, Henri Virtanen, Jussi Koivunen

**Affiliations:** 1 Department of Oncology and Radiotherapy Oulu University Hospital Oulu Finland; 2 Docrates Cancer Center Helsinki Finland; 3 Development Unit Hospital District of South-West Finland Turku Finland; 4 Kaiku Health Oy Helsinki Finland

**Keywords:** ePRO, immune checkpoint inhibitors, symptoms, side-effects, anti-PD-(L)1 therapy

## Abstract

**Background:**

Immune checkpoint inhibitors (ICIs) have become a standard of care for various tumor types. Their unique spectrum of side effects demands continuous and long-lasting assessment of symptoms. Electronic patient-reported outcome (ePRO) follow-up has been shown to improve survival and quality of life of cancer patients treated with chemotherapy.

**Objective:**

This study aimed to investigate whether ePRO follow-up of cancer patients treated with ICIs is feasible. The study analyzed (1) the variety of patient reported symptoms, (2) etiology of alerts, (3) symptom correlations, and (4) patient compliance.

**Methods:**

In this prospective, one-arm, multi-institutional study, we recruited adult cancer patients whose advanced cancer was treated with anti-programmed cell death protein 1 (PD)- ligand (L)1 agents in outpatient settings. The ePRO tool consisted of a weekly questionnaire evaluating the presence of typical side effects, with an algorithm assessing the severity of the symptom according to National Cancer Institute Common Terminology Criteria for Adverse Events and an urgency algorithm sending alerts to the care team. A patient experience survey was conducted monthly. The patients were followed up to 6 months or until disease progression.

**Results:**

A total of 889 symptom questionnaires was completed by 37 patients (lung cancer, n=15; melanoma, n=9; genitourinary cancer, n=9; head and neck cancer, n=4). Patients showed good adherence to ePRO follow-up. The most common grade 1 symptoms were fatigue (28%) and itching (13%), grade 2 symptoms were loss of appetite (12%) and nausea (12%), and grade 3-4 symptoms were cough (6%) and loss of appetite (4%). The most common reasons for alerts were loss of appetite and shortness of breath. In the treatment benefit analysis, positive correlations were seen between clinical benefit and itching as well as progressive disease and chest pain.

**Conclusions:**

According to the results, ePRO follow-up of cancer patients receiving ICIs is feasible. ePROs capture a wide range of symptoms. Some symptoms correlate to treatment benefit, suggesting that individual prediction models could be generated.

**Trial Registration:**

Clinical Trials Register, NCT3928938; https://clinicaltrials.gov/ct2/show/NCT03928938

## Introduction

Cancer patients experience a variety of symptoms derived from the malignancy itself as well as side effects of the given treatment. Many symptoms are left unnoticed due to factors such as limited symptom follow-up between prescheduled health care visits, nonsystematic evaluation of symptoms, and inadequate communication [1-7]. In general, worsening of symptoms indicates cancer progression or severe side effects of the treatment and is linked to poorer cancer survival [8].

Patient-reported outcomes (PROs) consist of health-related questionnaires completed by the patients themselves, which can capture symptoms and signs and their severity. Web-based reporting of PROs has many advantages compared to paper questionnaires such as reducing time to complete and overcoming geographic location limitations. Scheduled electronic patient-reported outcomes (ePROs) enable timely and continuous collection of symptoms in a cost-effective manner [9-14]. Furthermore, use of ePROs in cancer patient monitoring has shown impressive improvements in overall survival compared to standard follow-up [15,16]. In addition, ePROs can be coupled to an urgency algorithm, which sends an alert to the care unit upon report of severe or altering symptoms by a patient. This enables rapid reaction to and treatment of important medical events.

In the past 5 years, there has been significant advancement in the development of cancer immunotherapies with the introduction of immune checkpoint inhibitor (ICI) therapies such as anti-PD-(L)1 and anti-T-lymphocyte-associated protein 4 (CTLA-4) antibodies [17]. ICI therapies have become the most important medical therapies in many malignancies such as melanoma, non-small cell lung cancer, and urogenital cancers [18-27]. ICIs differ from traditional cancer therapies due to potentially severe side effects in all organs of the body and late timing of side effect occurrence [27-29]. Therefore, there is a need for comprehensive and ongoing assessment of symptoms.

Approximately 15% of patients receiving ICI monotherapies reportedly have severe grade 3-4 side effects, and about 30% have lower grade adverse events (AEs). Even life-threatening side effects can occur, but they can, in most cases, be managed with early detection, by delaying or stopping the ICI therapy, and with the initiation of immunosuppressive medication [30-32].

To our knowledge, this is the first prospective trial investigating ePROs in the follow-up of cancer patients receiving ICIs. The study aim was to investigate the feasibility of ePRO symptom follow-up and to analyze the spectrum of patient-reported symptoms, number and aetiology of urgency algorithm alerts, correlations between different symptoms and treatment benefit, and patient compliance.

## Methods

### Study Design and Participants

KISS was an investigator-initiated, multicenter, prospective, one-arm study, which was undertaken in 3 multidisease cancer centers in Finland. Patients were recruited during routine doctors’ appointments at study centers by study doctors. The inclusion criteria included advanced cancer to be treated with anti-PD-(L)1 in outpatient settings, initiation of anti-PD-(L)1 therapy had occurred ≤2 weeks prior to study recruitment, age ≥18 years, Eastern Cooperative Oncology Group ≤3, and availability of internet access and email. Baseline information such as basic laboratory values, age, and gender were collected from electronic health care records. After providing written informed consent, study patients received a short (5-15 minutes) instruction on how to use the Kaiku software by a study physician. At the initiation of the treatment phase (within 0-2 weeks from the first anti-PD-(L)1 infusion) and weekly thereafter until treatment discontinuation or 6 months of follow-up, patients received an email notification to complete the baseline electronic symptom questionnaire of 17 questions. If a weekly symptom questionnaire was not completed on the day of email receipt, daily email reminders were sent for 6 days. In addition, patients were asked to fill in a monthly electronic patient experience survey until treatment discontinuation or 6 months of follow-up. The use of the ePRO tool was free of charge for the patients and study centers. Online technical support by Kaiku Health for the users was available from 8 am to 4 pm Monday to Friday. The investigators evaluated the treatment response according to Response Evaluation Criteria in Solid Tumors (RECIST) 1.1 criteria at 8-10 weeks after treatment initiation. Clinical benefit rate was selected as a benefit measure instead of objective response rate since (1) we had a small number of study subjects, (2) responses were analyzed only up to 12 weeks from inclusion, and (3) the correlation between clinical benefit and objective response rate is not as clearly defined with immunotherapies as with traditional cancer medications.

According to the protocol, study results were analyzed when the last included patient had 12 weeks of follow-up. The major endpoints of the study included (1) patient-reported symptoms and their severity; (2) number of triggered alerts by the ePRO tool and their correlation to treatment side effects, cancer progression, other medical events, or survival; (3) correlations between different symptoms and treatment side effects, cancer progression, other medical events, or survival; and (4) patient compliance using the patient experience survey and response rates to symptom questionnaires. Sample size was based on the estimation that 15% of patients receiving ICI monotherapies will experience severe (grade 3-4) side effects and about 30% will experience lower grade AE. In a 40-patient cohort, 3-6 patients will experience a severe immune-related AE. It was estimated that the expected study population is sufficient to evaluate the feasibility of the symptom questionnaire in detecting severe AEs. Questionnaires from several timepoints were estimated to be collected from 90% of the study population (~35 patients), which would enable a more comprehensive assessment of feasibility, patient experience, and correlation of ePRO changes to treatment response and survival.

All data collection was carried out according to national legislation and under permit from the medical director of each research center. The study was approved by the Pohjois-Pohjanmaan sairaanhoitopiiri (PPSHP) ethics committee (number 9/2017), Valvira (number 361), and Oulu University Hospital Ethics Committee (9/2017). The study was conducted in accordance with the Declaration of Helsinki and Good Clinical Practice guidelines.

### ePRO Follow-Up

The Kaiku Health ePRO tool is a web-based solution scaled to be used easily on smartphones and home computers. The Kaiku Health immune-oncology module designed for the study consists of 17 questions. The symptoms selected for the Kaiku Health symptom tracking tool for cancer immunotherapy are based on the most common AEs that have occurred during clinical trials of anti-PD-1, anti-PD-L1, and anti-CTLA-4 monotherapies. The symptoms tracked by the instrument are potential signs and symptoms of immune-related AEs. The symptom selection was based on publications from the following clinical trials: CheckMate 017 (NCT01642004), CheckMate 026 (NCT02041533), CheckMate 057 (NCT01673867), CheckMate 066 (NCT01721772), CheckMate 067 (NCT01844505), KEYNOTE-010 (NCT01905657), and OAK (NCT02008227). Food and Drug Administration labels for nivolumab, pembrolizumab, and atezolizumab were also used in the symptom selection for the instrument. The questions for each symptom in the instrument were developed based on the National Cancer Institute Common Terminology Criteria for Adverse Events (NCI-CTCAE) register by converting the description of a grading into patient-friendly language. Any criterion that would be impossible for patients to report has been excluded from the available questions. Developing the symptom questionnaire in this manner enabled self-reporting by patients and development of an algorithm that provides an assessment and approximation of the severity of each symptom according to NCI-CTCAE criteria. NCI-CTCAE grades the symptoms from 0 to 4: no (0), mild (1), moderate (2), severe (3), and life-threatening (4).

Questions assess the presence of blood in stool, blood in urine, blurred vision, chest pain, cough, loss of appetite, diarrhea, dizziness, fatigue, fever, headache, itching, nausea, pain in joints, rash, shortness of breath, stomach pain, and vomiting. Besides recording the presence of a symptom, a severity algorithm that grades the symptom according to NCI-CTCAE was applied. The severity algorithm triggered an email alert to the study physician of the care unit based on preset limits (presence of a grade 3 or higher symptom or increase in symptom severity from grade 0 to 2). The patients were informed that the care unit would react to the alerts promptly within 3 days; thus, the ePRO follow-up was intended only for nonurgent communication, and in urgent matters, patients were advised to contact emergency care.

### Patient Experience Survey

Study participants were requested to reply to a monthly patient experience survey. The patient experience survey consisted of 6 yes/no or multiple-choice questions. The survey was developed by the investigators for the study and has not been previously validated.

### Statistical Analysis

The analysis was carried out when the last patient included had 12 weeks of follow-up data available. Correlations of different patient-reported symptoms were analyzed using heat maps with Pearson product-moment correlation. In the heat map analysis, the intensity of the color signifies the level of correlation: red, negative correlation; blue, positive correlation. In other words, a large effect correlation was defined as 0.5; medium as 0.3, and small as 0.1 (absolute values).

## Results

### Study Accrual and Patient Characteristics

**Figure 1:**
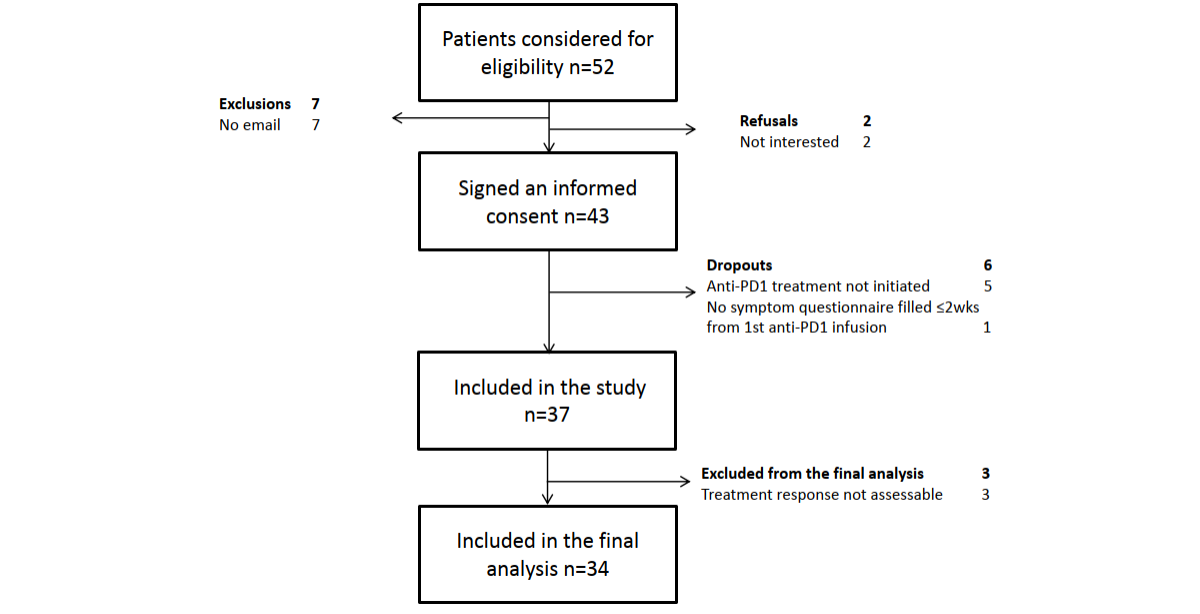
Flowchart of patient accrual and analysis.

Patient recruitment took place between June 2017 and March 2019, and the last study patient visit was in June 2019. Anticipated recruitment for the study was 40 patients in 12 months, but due to a slow recruiting pace, the period was extended. Informed consent was provided by 43 patients, and analysis was limited to a total of 37 patients who had anti-PD(L)-1 therapy initiated and answered at least 2 symptom questionnaires (baseline and one following; Figure 1). No technical issues nor security breaches related to the web-based tool occurred during the study period.

The median age of the study participants was 62 years (range 32-80 years). The majority of patients were male (27/37, 73%), and 5 patients had a history of an autoimmune disease, with hypothyreosis (4/5, 80%) being the most common. Tumor types included lung cancer (15/37, 41%), melanoma (9/37, 24%), genitourinary (GU) cancer (9/37, 24%), and head and neck cancer (4/37, 11%), and 28 (28/37, 76%) patients had stage IV disease (Table 1).

**Table 1:** Patient demographics.

Characteristics		Results
Age (years), median		61.7
**Gender, n (%)**		
	Male	27 (73)
	Female	10 (27)
**Autoimmune disease, n (%)**		
	Yes	5 (14)
	No	32 (87)
**Tumor type, n (%)**		
	Melanoma	9 (24)
	Lung cancer	15 (41)
	Genitourinary cancer	9 (24)
	Head and neck	4 (11)
**Stage at diagnosis, n (%)**		
	Stage III	9 (24)
	Stage IV	28 (76)
**Eastern Cooperative Oncology Group (** **ECOG), n (%)**		
	0	20 (54)
	1	15 (41)
	2	2 (5)

### Patient-Reported Symptoms and Alerts

During the study, 889 completed symptom questionnaires were registered. The range of answered questionnaires was 0.583-1.27 per patient per week, with high response rates throughout the complete follow-up period up to 24 weeks (Table 2).

**Table 2:** Average number of answered symptom questionnaires completed per patient per week, up to 24 weeks.

Week	Number of questionnaires per patient, mean
1	1.27
2	0.882
3	1.14
4	0.861
5	1.06
6	0.833
7	0.833
8	0.861
9	0.765
10	0.842
11	0.882
12	0.748
13	0.991
14	0.824
15	0.742
16	0.707
17	0.704
18	0.719
19	0.733
20	0.826
21	0.83
22	0.611
23	0.583
24	0.798

During the first 12 weeks of ePRO follow-up, the most common grade 1-2 symptoms were fatigue (346/889, 39%), cough (187/889, 21%), pain in joints (160/889, 18%), itching (151/889, 17%), loss of appetite (151/889, 17%), nausea (151/889, 17%), and shortness of breath (133/889, 15%). The most common grade 3-4 symptoms were cough (53/889, 6%), loss of appetite (36/889, 4%), and nausea (36/889, 4%). None of the patients (0/37) reported blood in stool or hematuria (Table 3).

**Table 3:** Distribution of the severity of the reported symptoms according to all the answered symptom questionnaires (n=889) in weeks 1-12.

Symptom	Grade 0, %	Grade 1, %	Grade 2, %	Grades 3-4, %
Blood in stool	100	0	0	0
Blurred vision	96	0	4	0
Chest pain	94	4	1	1
Cough	74	12	9	6
Diarrhea	96	3	1	0
Dizziness	92	6	2	0
Fatigue	60	28	11	1
Fever	95	5	0	0
Headache	87	10	2	0
Hematuria	100	0	0	0
Itching	83	13	4	1
Loss of appetite	79	5	12	4
Nausea	94	5	12	4
Pain in joints	81	12	6	2
Rash	88	9	1	1
Shortness of breath	83	8	7	2
Stomach pain	94	3	2	1
Vomiting	98	2	0	0

Of the 391 answered symptom questionnaires during the first 12 weeks, the ePRO tool triggered 67 (67/391, 17.1%) alerts. The most common reasons for alerts were loss of appetite, shortness of breath, pain in joints, blurred vision, and cough. The treating physicians were asked to evaluate the etiology of alerts by grading them to cancer, treatment, or unclear categories. Unclear reasons were the most common cause of alerts (38/67, 57%), followed by treatment (21/67, 31%) and cancer (8/67, 11%; Table 4).

**Table 4:** Etiology of the symptom questionnaire alerts (n=67).

Characteristics		n (%)
**Etiology**		
	Unclear	38 (57)
	Treatment	21 (31)
	Cancer	8 (11)
**By symptom**		
	Loss of appetite	32 (48)
	Shortness of breath	31 (46)
	Pain in joints	21 (31)
	Blurred vision	17 (25)
	Cough	16 (24)
	Fatigue	15 (22)
	Itching	12 (18)
	Chest pain	9 (13)
	Headache	8 (12)
	Stomach pain	6 (9)
	Rash	6 (9)
	Nausea	5 (8)
	Diarrhea	3 (5)
	Dizziness	3 (5)

### Patient Compliance

Patient compliance was assessed every 4 weeks based on the electronic patient experience survey provided through Kaiku software. During the first 12 weeks, 31 patients replied to the survey, and analysis was limited to these. All the patients replied that using the Kaiku software was easy or very easy, and only 1 of 6 patients reported that they needed assistance using the software. Over 90% of the patients (29/31, 94%) reported that the questions were understandable. In addition, 90% of the patients (28/31, 90%) felt that the Kaiku ePRO follow-up improved their cancer care, and 95% (29/31) said they would recommend using it in the follow-up of cancer patients (Table 5).

**Table 5:** Kaiku Health patient experience survey results during the first 12 weeks of follow-up (n=31).

Survey questions		n (%)
**How easy or difficult is the use of the Kaiku Health application?**		
	Very easy	15 (48)
	Easy	16 (52)
	Difficult	0
	Very difficult	0
	I cannot say	0
**Have you needed the help of another person to use the Kaiku Health application, not taking into account the training that you received at the health care unit?**		
	Yes	5 (16)
	No	26 (84)
**Were the questions in the symptom questionnaire in the Kaiku Health application understandable?**		
	Totally agree	21 (68)
	Partly agree	8 (26)
	Partly disagree	2 (7)
	Totally disagree	0
	I cannot say	0
**Do you think that the use of the Kaiku Health application will improve the follow-up of your cancer treatment (compared to a situation where the application would not have been used)?**		
	Yes	28 (90)
	No	3 (10)
	I cannot say	0
**Have you benefited from using the Kaiku Health application?**		
	Yes	19 (61)
	No	1 (3)
	I cannot say	11 (36)
**Would you recommend the use of the Kaiku Health application in cancer care follow-up?**		
	Yes	29 (94)
	No	0
	I cannot say	2 (7)

### Correlations Between Patient-Reported Symptoms and Treatment Benefit

**Figure 2:**
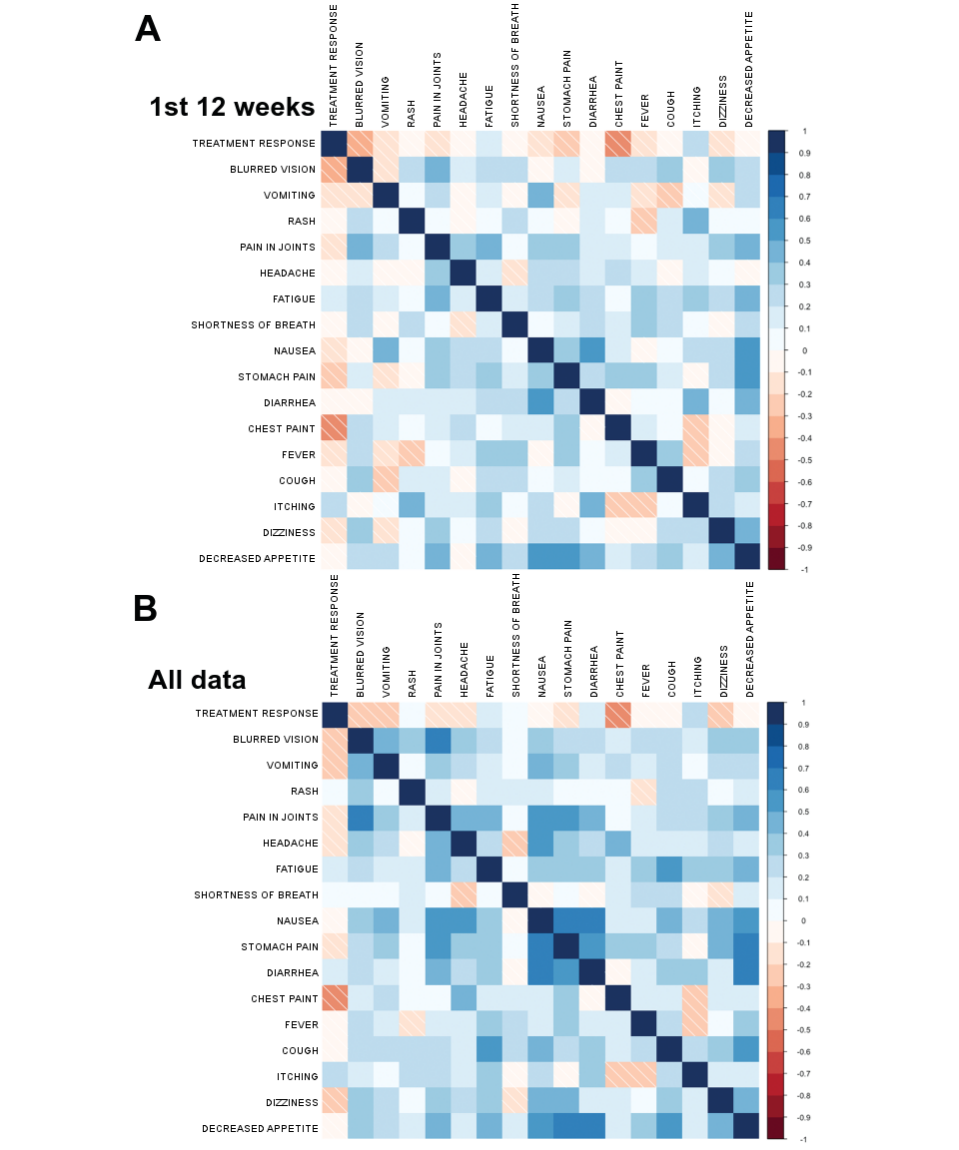
Correlation analysis between different symptoms and treatment benefit (complete response, partial response, or stable disease as a best response) using heat maps during the (A) first 12 weeks of follow-up and (B) entire study. The color intensity signifies the correlation strength (0.5, large effect; 0.3, medium effect; 0.1, small effect): red, negative correlation; blue, positive correlation.

Correlations between ePRO-collected symptoms were analyzed using heat maps. According to the results, the symptom correlations during the first 12 weeks and beyond were very similar (Figure 2). During the first 12 weeks, large positive correlations were seen between nausea, diarrhea, loss of appetite, and vomiting; stomach pain and decreased appetite; and rash and itching. Only small negative correlations were detected between cough and vomiting, itching and chest pain, and itching and fever (Figure 2A).

**Figure 3:**
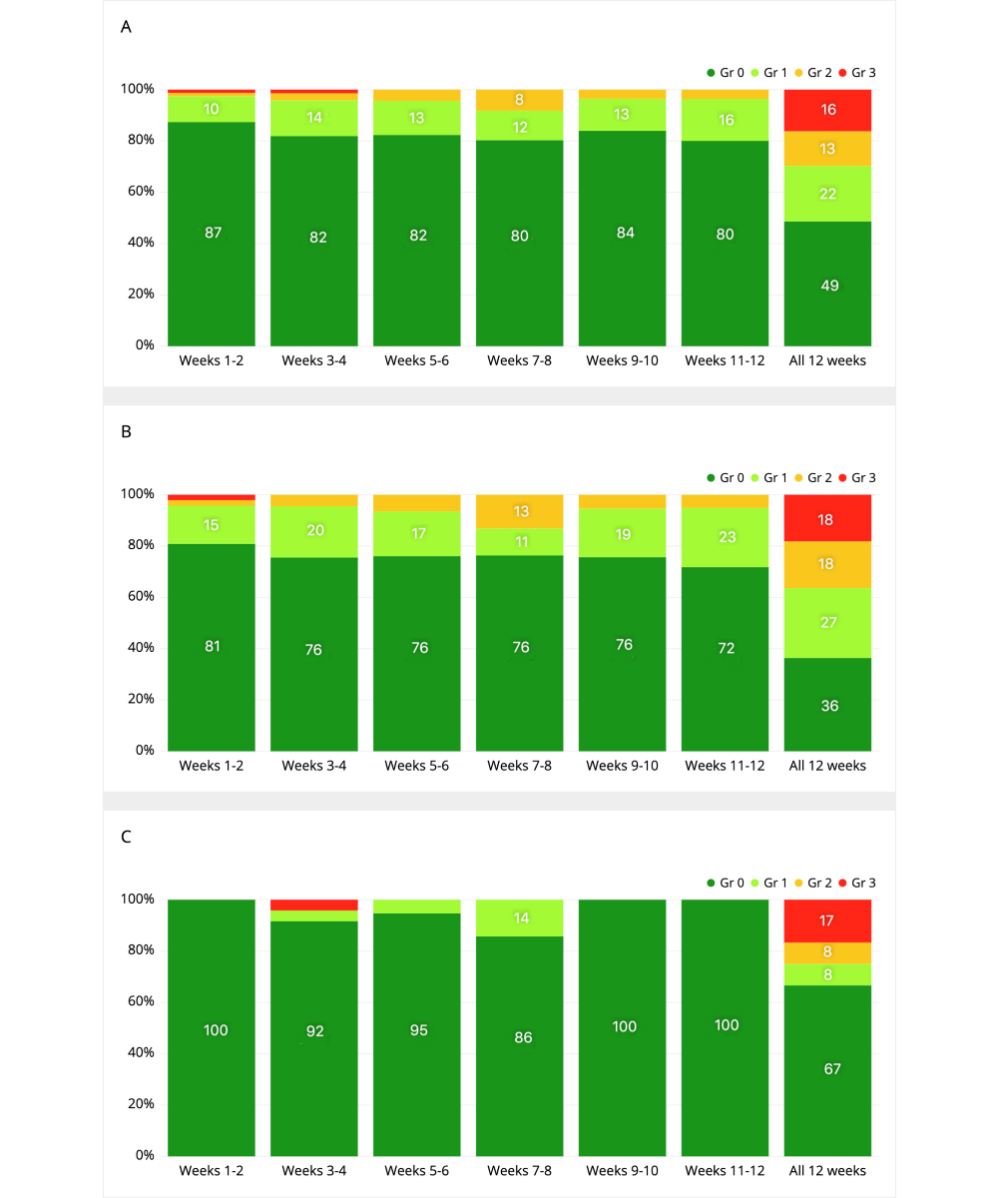
Distribution of the symptom grades reported on the symptom questionnaires during the first 12 weeks for itching for (A) all patients, (B) patients with complete response (CR)/partial response (PR)/stable disease (SD), and (C) patients with progressive disease (PD).

**Figure 4:**
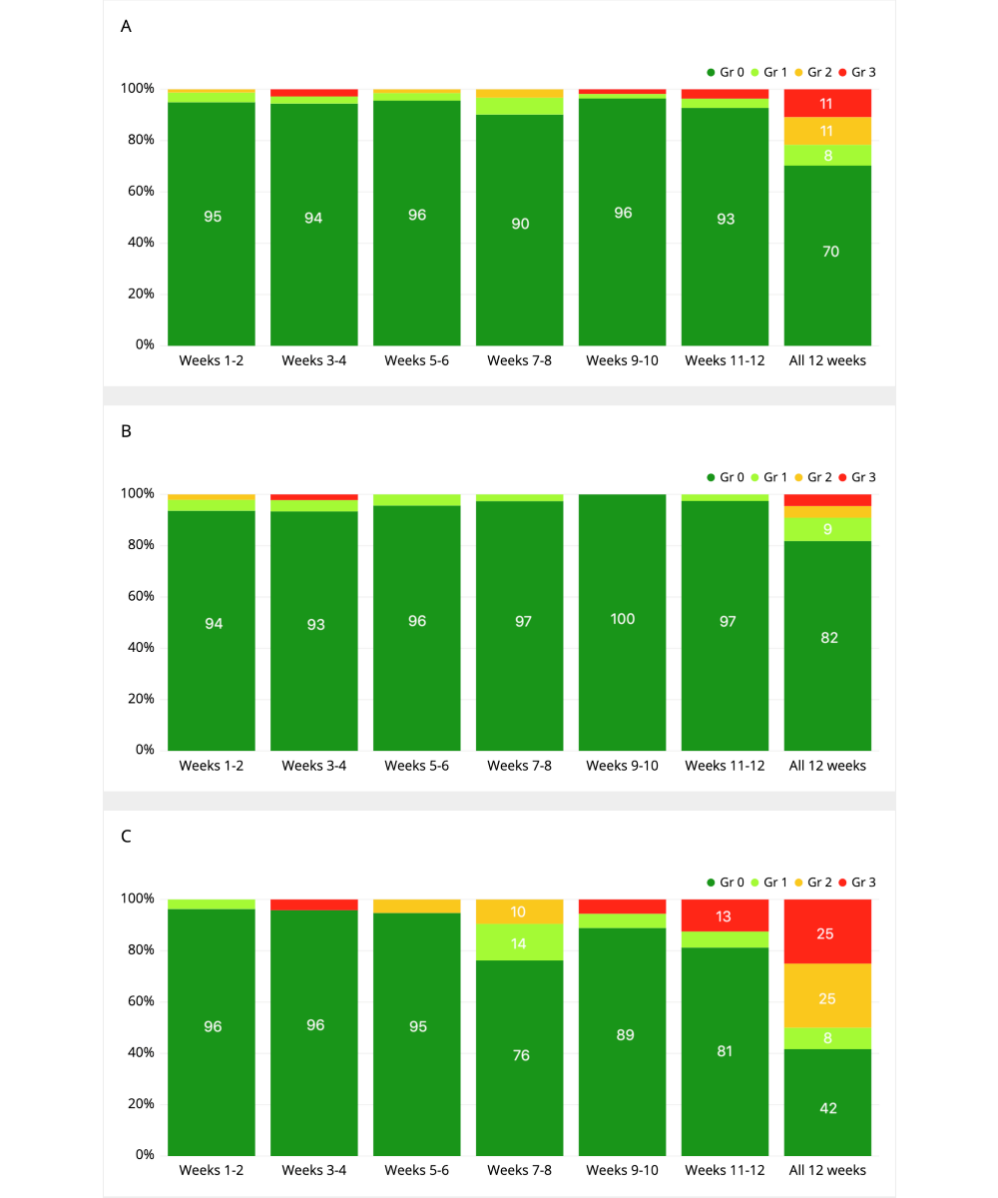
Distribution of the symptom grades reported on the symptom questionnaires during the first 12 weeks for chest pain for (A) all patients, (B) patients with complete response (CR)/partial response (PR)/stable disease (SD), and (C) patients with progressive disease (PD).

Of the 37 patients, 34 were evaluated for objective treatment response (RECIST 1.1) by the investigators and included in the treatment benefit analysis. Of the 34 patients, 22 (65%) patients had complete response (CR), partial response (PR), or stable disease (SD) as the best response, while 12 (12/34, 35%) patients had progressive disease (PD). The heat map analysis suggested a small positive correlation between clinical benefit (CR/PR/SD) and itching (0.23 for the first 12 weeks, Figure 2A; 0.25 for all data, Figure 2B) and medium correlations between PD and chest pain (–0.41 for the first 12 weeks, Figure 2A; –0.47 for all data, Figure 2B). We further analyzed symptom progression and severity for itching and chest pain. During the first 12 weeks, 15-23% of the patients with clinical benefit reported itching, while the rate was much lower for patients with PD (0-14%; Figure 3). Furthermore, the average grade was much higher for patients with clinical benefit (weeks 1-12, 0.26-0.37; all 12 weeks, 1.18) compared to patients with PD (weeks 1-12, 0-0.17; all 12 weeks, 0.75; Table 6). For the complete follow-up period, most of the patients with clinical benefit had itching (14/22, 64%), while this was much lower for patients with PD (4/12, 33%; Figure 3). The severity of itching for the patients with clinical benefit was mainly low grade (grade 1: 6/22, 27%; grade 2: 4/22, 18%; Figure 3). During the complete follow-up period, chest pain was much more common in patients with PD (7/12, 58%) than in the patients with clinical benefit (4/22, 18%; Figure 4). In the first 12 weeks, patients with PD had a tendency for gradually increasing average grades for chest pain; conversely, a continuing decrease in the average grade was seen for patients who responded to the therapy (Table 6).

**Table 6:** Average grade of itching and chest pain reported by patients.

Weeks	Itching			Chest pain			
	Entire sample (n=34)	CR^a^/PR^b^/SD^c^ (n=22)	PD^d^ (n=12)		Entire sample (n=34)	CR/PR/SD (n=22)	PD (n=12)
Weeks 1-2	0.16	0.26	0.00		0.06	0.09	0.04
Weeks 3-4	0.24	0.29	0.17		0.11	0.11	0.13
Weeks 5-6	0.22	0.30	0.05		0.06	0.04	0.11
Weeks 7-8	0.28	0.37	0.14		0.13	0.03	0.33
Weeks 9-10	0.20	0.30	0.00		0.07	0.00	0.22
Weeks 11-12	0.24	0.33	0.00		0.15	0.03	0.44
All 12 weeks	0.97	1.18	0.75		0.62	0.32	1.33

^a^CR: complete response.

^b^PR: partial response.

^c^SD: stable disease.

^d^PD: progressive disease.

## Discussion

According to previous studies, ePRO follow-up has improved survival and quality of life compared to routine surveillance when used with cancer patients receiving chemotherapy and lung cancer patients treated with curative intention [15,16]. However, the ePRO approach remains virtually unstudied in the context of cancer immunotherapies [33]. For optimal follow-up of patients receiving ICIs, there is a need for comprehensive assessment, grading, and long-term surveillance of symptoms. ePROs could provide a cost-effective follow-up tool to meet these 3 requirements. We previously reported a retrospective pilot study of ePRO follow-up of cancer patients treated with ICIs [34]. To our knowledge, this study is the first prospective clinical trial investigating ePRO follow-up of cancer patients treated with anti-PD-(L)1 therapies.

In this study, we used an ePRO module with 17 questions and an algorithm grading the PROs according to NCI-CTCAE. The questionnaire was designed specifically for patients receiving ICIs based on the published side effect profile of these agents. The symptom variety based on patient reporting and the grading algorithm performed well, and the symptom data followed closely what has been reported in clinical trials investigating ICIs. A recent meta-analysis with more than 20,000 patients suggested that fatigue (18%), itching (11%), and diarrhea (9%) are the most common AEs reported in patients treated with anti-PD-(L)1 agents [35]. The incidence of AEs in clinical trials are generally lower than in our study, which might be related to better capture of patient-reported low-grade symptoms, which are often overlooked in physician-based AE reporting in clinical trials [36-42].

In the present study, the symptom questionnaire was also coupled to an urgency algorithm, which generated alerts in 17% of the answered questionnaires during the first 12 weeks. Loss of appetite and fatigue were among the most common symptoms generating alerts. These symptoms very rarely alter the cancer treatment, and symptomatic treatments are scarce. Furthermore, physicians determined that most of the alerts were caused by unclear reasons, which is probably related to the high frequency of symptoms with unclear etiology. Fine-tuning of the alerts to focus not only on the symptom grade but also the nature of the symptom could lower the number of alerts and staff workload without sacrificing the performance of ePROs.

Patient adherence to and experience with ePRO follow-up was found to be very good in this study. The patients were requested by email to complete symptom questionnaires weekly, and the number of completed questionnaires was very close to one per patient per week for the first 12 weeks. Based on the patient experience surveys, the system was easy to use, and patients felt that ePRO follow-up improved their cancer care, which is in line with previous studies [40,41].

Our previous retrospective study with patients treated with ICIs suggested that some ePRO-reported dermatological, gastrointestinal (GI), and pulmonary symptoms co-occur [34]. Similarly, we saw large positive correlations between treatment response and GI symptoms as well as between treatment response and dermatological symptoms. Furthermore, the data showed small negative correlations between pulmonary symptoms and some GI symptoms and between itching, pulmonary symptoms, and fever. In our previous retrospective study, which did not include data on the treatment responses, we generated a hypothesis that GI and skin symptoms might be related to immune activation and treatment benefit, while pulmonary symptoms could signal tumor progression. Since this study also included data on treatment benefit, it enabled us to investigate our hypothesis. The results showed that there was a small positive correlation between treatment benefit (CR/PR/SD) and itching (ePRO) and between PD and chest pain (ePRO). Previous studies have linked autoimmune skin toxicity (rash) to PD-1 agent benefit [43-45]. Our results are hypothesis-generating while suggesting that ePRO-collected symptom data can mimic physician-assessed symptoms and correlate with treatment benefit. Furthermore, compared to physician-based AE reporting, it is possible that ePROs enable enhanced capturing of low-grade AEs without visible presentation such as itching and therefore facilitate predicting clinical treatment benefit.

ePROs enable cost-effective capture of symptoms and their change over long periods [46]. Changes over time might better predict treatment side effects and benefit than just a single presentation of a symptom. Furthermore, data from this study showed that early (in the first 12 weeks) changes in symptoms correlate with treatment benefit as well as symptoms from the whole follow-up period. This further highlights the possibility that early changes in symptoms predict outcomes. Large-scale symptom data coupled with treatment benefit and side effects could be used to build prediction models using artificial intelligence methods. These models could predict an individual’s risk for symptom development, treatment-related side effects, and treatment benefit.

Our study has some limitations. The sample size is small (n=37); however, the size is typical for feasibility studies. The small sample size prevents us from making strong generalizations based on the data. The one-arm design of the study precludes comparison of the effectiveness of the intervention. However, we feel that our study is important since it lays the groundwork for future studies on the topic.

In conclusion, this study is the first reported prospective clinical trial investigating the use of ePROs in the follow-up of cancer patients treated with ICIs. The results of this study suggest that follow-up of cancer patients using ePROs is feasible, enabling comprehensive capturing of symptoms over long periods with good patient adherence and satisfaction. Moreover, some early patient-reported symptoms were found to correlate with treatment benefit suggesting that individual prediction models for treatment benefit could be generated.

## Appendix

Multimedia Appendix 1 CONSORT EHEALTH checklist (V 1.6.1).

## Abbreviations

AE: adverse event
CTLA-4: cytotoxic T-lymphocyte-associated protein 4
CR: complete response
ECOG: Eastern Cooperative Oncology Group
ePRO: electronic patient-reported outcome
GI: gastrointestinal
ICI: immune checkpoint inhibitor
NCI-CTCAE: National Cancer Institute Common Terminology Criteria for Adverse Events
PD: progressive disease
PD-(L)1: protein 1-ligand 1
PR: partial response
PRO: patient-reported outcome
RECIST: Response Evaluation Criteria in Solid Tumors
SD: stable disease

## References

[1] Basch E, Jia X, Heller G, Barz A, Sit L, Fruscione M, Appawu M, Iasonos A, Atkinson T, Goldfarb S, Culkin A, Kris MG, Schrag D (2009). Adverse symptom event reporting by patients vs clinicians: relationships with clinical outcomes. J Natl Cancer Inst.

[2] Gilbert JE, Howell D, King S, Sawka C, Hughes E, Angus H, Dudgeon D (2012). Quality improvement in cancer symptom assessment and control: the Provincial Palliative Care Integration Project (PPCIP). J Pain Symptom Manage.

[3] Henry DH, Viswanathan HN, Elkin EP, Traina S, Wade S, Cella D (2008). Symptoms and treatment burden associated with cancer treatment: results from a cross-sectional national survey in the U.S. Support Care Cancer.

[4] Laugsand EA, Sprangers MA, Bjordal K, Skorpen F, Kaasa S, Klepstad P (2010). Health care providers underestimate symptom intensities of cancer patients: a multicenter European study. Health Qual Life Outcomes.

[5] Reilly CM, Bruner DW, Mitchell SA, Minasian LM, Basch E, Dueck AC, Cella D, Reeve BB (2013). A literature synthesis of symptom prevalence and severity in persons receiving active cancer treatment. Support Care Cancer.

[6] Valderas JM, Kotzeva A, Espallargues M, Guyatt G, Ferrans CE, Halyard MY, Revicki DA, Symonds T, Parada A, Alonso J (2008). The impact of measuring patient-reported outcomes in clinical practice: a systematic review of the literature. Qual Life Res.

[7] Velikova G, Keding A, Harley C, Cocks K, Booth L, Smith AB, Wright P, Selby PJ, Brown JM (2010). Patients report improvements in continuity of care when quality of life assessments are used routinely in oncology practice: secondary outcomes of a randomised controlled trial. Eur J Cancer.

[8] Trajkovic-Vidakovic M, de Graeff A, Voest EE, Teunissen SC (2012). Symptoms tell it all: a systematic review of the value of symptom assessment to predict survival in advanced cancer patients. Crit Rev Oncol Hematol.

[9] Bennett AV, Jensen RE, Basch E (2012). Electronic patient-reported outcome systems in oncology clinical practice. CA Cancer J Clin.

[10] Cleeland CS, Wang XS, Shi Q, Mendoza TR, Wright SL, Berry MD, Malveaux D, Shah PK, Gning I, Hofstetter WL, Putnam JB, Vaporciyan AA (2011). Automated Symptom Alerts Reduce Postoperative Symptom Severity After Cancer Surgery: A Randomized Controlled Clinical Trial. JCO.

[11] Holch P, Warrington L, Bamforth L, Keding A, Ziegler L, Absolom K, Hector C, Harley C, Johnson O, Hall G, Morris C, Velikova G (2017). Development of an integrated electronic platform for patient self-report and management of adverse events during cancer treatment. Ann Oncol.

[12] Jensen RE, Snyder CF, Abernethy AP, Basch E, Potosky AL, Roberts AC, Loeffler DR, Reeve BB (2014). Review of electronic patient-reported outcomes systems used in cancer clinical care. J Oncol Pract.

[13] Kotronoulas G, Kearney N, Maguire R, Harrow A, Di Domenico D, Croy S, MacGillivray S (2014). What is the value of the routine use of patient-reported outcome measures toward improvement of patient outcomes, processes of care, and health service outcomes in cancer care? A systematic review of controlled trials. J Clin Oncol.

[14] Pakhomov S, Jacobsen S, Chute C, Roger V (2008). Agreement between patient-reported symptoms and their documentation in the medical record. Am J Manag Care.

[15] Basch E, Deal AM, Dueck AC, Scher HI, Kris MG, Hudis C, Schrag D (2017). Overall Survival Results of a Trial Assessing Patient-Reported Outcomes for Symptom Monitoring During Routine Cancer Treatment. JAMA.

[16] Denis F, Yossi S, Septans A, Charron A, Voog E, Dupuis O, Ganem G, Pointreau Y, Letellier C (2017). Improving Survival in Patients Treated for a Lung Cancer Using Self-Evaluated Symptoms Reported Through a Web Application. American Journal of Clinical Oncology.

[17] Topalian SL, Hodi FS, Brahmer JR, Gettinger SN, Smith DC, McDermott DF, Powderly JD, Carvajal RD, Sosman JA, Atkins MB, Leming PD, Spigel DR, Antonia SJ, Horn L, Drake CG, Pardoll DM, Chen L, Sharfman WH, Anders RA, Taube JM, McMiller TL, Xu H, Korman AJ, Jure-Kunkel M, Agrawal S, McDonald D, Kollia GD, Gupta A, Wigginton JM, Sznol M (2012). Safety, Activity, and Immune Correlates of Anti–PD-1 Antibody in Cancer. N Engl J Med.

[18] Bellmunt J, de Wit R, Vaughn DJ, Fradet Y, Lee J, Fong L, Vogelzang NJ, Climent MA, Petrylak DP, Choueiri TK, Necchi A, Gerritsen W, Gurney H, Quinn DI, Culine S, Sternberg CN, Mai Y, Poehlein CH, Perini RF, Bajorin DF (2017). Pembrolizumab as Second-Line Therapy for Advanced Urothelial Carcinoma. N Engl J Med.

[19] Borghaei H, Paz-Ares L, Horn L, Spigel DR, Steins M, Ready NE, Chow LQ, Vokes EE, Felip E, Holgado E, Barlesi F, Kohlhäufl M, Arrieta O, Burgio MA, Fayette J, Lena H, Poddubskaya E, Gerber DE, Gettinger SN, Rudin CM, Rizvi N, Crinò L, Blumenschein GR, Antonia SJ, Dorange C, Harbison CT, Graf Finckenstein F, Brahmer JR (2015). Nivolumab versus Docetaxel in Advanced Nonsquamous Non–Small-Cell Lung Cancer. N Engl J Med.

[20] Brahmer J, Reckamp KL, Baas P, Crinò L, Eberhardt WE, Poddubskaya E, Antonia S, Pluzanski A, Vokes EE, Holgado E, Waterhouse D, Ready N, Gainor J, Arén Frontera O, Havel L, Steins M, Garassino MC, Aerts JG, Domine M, Paz-Ares L, Reck M, Baudelet C, Harbison CT, Lestini B, Spigel DR (2015). Nivolumab versus Docetaxel in Advanced Squamous-Cell Non–Small-Cell Lung Cancer. N Engl J Med.

[21] Herbst RS, Baas P, Kim D, Felip E, Pérez-Gracia JL, Han J, Molina J, Kim J, Arvis CD, Ahn M, Majem M, Fidler MJ, de Castro G, Garrido M, Lubiniecki GM, Shentu Y, Im E, Dolled-Filhart M, Garon EB (2016). Pembrolizumab versus docetaxel for previously treated, PD-L1-positive, advanced non-small-cell lung cancer (KEYNOTE-010): a randomised controlled trial. The Lancet.

[22] Motzer RJ, Escudier B, McDermott DF, George S, Hammers HJ, Srinivas S, Tykodi SS, Sosman JA, Procopio G, Plimack ER, Castellano D, Choueiri TK, Gurney H, Donskov F, Bono P, Wagstaff J, Gauler TC, Ueda T, Tomita Y, Schutz FA, Kollmannsberger C, Larkin J, Ravaud A, Simon JS, Xu L, Waxman IM, Sharma P (2015). Nivolumab versus Everolimus in Advanced Renal-Cell Carcinoma. N Engl J Med.

[23] Reck M, Rodríguez-Abreu D, Robinson AG, Hui R, Csőszi T, Fülöp A, Gottfried M, Peled N, Tafreshi A, Cuffe S, O’Brien M, Rao S, Hotta K, Leiby MA, Lubiniecki GM, Shentu Y, Rangwala R, Brahmer JR (2016). Pembrolizumab versus Chemotherapy for PD-L1–Positive Non–Small-Cell Lung Cancer. N Engl J Med.

[24] Rittmeyer A, Barlesi F, Waterkamp D, Park K, Ciardiello F, von Pawel J, Gadgeel SM, Hida T, Kowalski DM, Dols MC, Cortinovis DL, Leach J, Polikoff J, Barrios C, Kabbinavar F, Frontera OA, De Marinis F, Turna H, Lee J, Ballinger M, Kowanetz M, He P, Chen DS, Sandler A, Gandara DR (2017). Atezolizumab versus docetaxel in patients with previously treated non-small-cell lung cancer (OAK): a phase 3, open-label, multicentre randomised controlled trial. The Lancet.

[25] Schachter J, Ribas A, Long GV, Arance A, Grob J, Mortier L, Daud A, Carlino MS, McNeil C, Lotem M, Larkin J, Lorigan P, Neyns B, Blank C, Petrella TM, Hamid O, Zhou H, Ebbinghaus S, Ibrahim N, Robert C (2017). Pembrolizumab versus ipilimumab for advanced melanoma: final overall survival results of a multicentre, randomised, open-label phase 3 study (KEYNOTE-006). The Lancet.

[26] Robert C, Schachter J, Long GV, Arance A, Grob JJ, Mortier L, Daud A, Carlino MS, McNeil C, Lotem M, Larkin J, Lorigan P, Neyns B, Blank CU, Hamid O, Mateus C, Shapira-Frommer R, Kosh M, Zhou H, Ibrahim N, Ebbinghaus S, Ribas A (2015). Pembrolizumab versus Ipilimumab in Advanced Melanoma. N Engl J Med.

[27] Weber JS, Hodi FS, Wolchok JD, Topalian SL, Schadendorf D, Larkin J, Sznol M, Long GV, Li H, Waxman IM, Jiang J, Robert C (2017). Safety Profile of Nivolumab Monotherapy: A Pooled Analysis of Patients With Advanced Melanoma. JCO.

[28] Li H, Ma W, Yoneda KY, Moore EH, Zhang Y, Pu LLQ, Frampton GM, Molmen M, Stephens PJ, Li T (2017). Severe nivolumab-induced pneumonitis preceding durable clinical remission in a patient with refractory, metastatic lung squamous cell cancer: a case report. J Hematol Oncol.

[29] Topalian SL, Sznol M, McDermott DF, Kluger HM, Carvajal RD, Sharfman WH, Brahmer JR, Lawrence DP, Atkins MB, Powderly JD, Leming PD, Lipson EJ, Puzanov I, Smith DC, Taube JM, Wigginton JM, Kollia GD, Gupta A, Pardoll DM, Sosman JA, Hodi FS (2014). Survival, Durable Tumor Remission, and Long-Term Safety in Patients With Advanced Melanoma Receiving Nivolumab. JCO.

[30] Haanen J, Carbonnel F, Robert C, Kerr K, Peters S, Larkin J, Jordan K, ESMO Guidelines Committee (2017). Management of toxicities from immunotherapy: ESMO Clinical Practice Guidelines for diagnosis, treatment and follow-up. Ann Oncol.

[31] Puzanov I, Diab A, Abdallah K, Bingham CO, Brogdon C, Dadu R, Hamad L, Kim S, Lacouture ME, LeBoeuf NR, Lenihan D, Onofrei C, Shannon V, Sharma R, Silk AW, Skondra D, Suarez-Almazor ME, Wang Y, Wiley K, Kaufman HL, Ernstoff MS, Society for Immunotherapy of Cancer Toxicity Management Working Group (2017). Managing toxicities associated with immune checkpoint inhibitors: consensus recommendations from the Society for Immunotherapy of Cancer (SITC) Toxicity Management Working Group. J Immunother Cancer.

[32] Spain L, Diem S, Larkin J (2016). Management of toxicities of immune checkpoint inhibitors. Cancer Treat Rev.

[33] Denis F, Lethrosne C, Pourel N, Molinier O, Pointreau Y, Domont J, Bourgeois H, Senellart H, Trémolières P, Lizée T, Bennouna J, Urban T, El Khouri C, Charron A, Septans AL, Balavoine M, Landry S, Solal-Céligny P, Letellier C (2017). Randomized Trial Comparing a Web-Mediated Follow-up With Routine Surveillance in Lung Cancer Patients. J Natl Cancer Inst.

[34] Iivanainen S, Alanko T, Peltola K, Konkola T, Ekström J, Virtanen H, Koivunen JP (2019). ePROs in the follow-up of cancer patients treated with immune checkpoint inhibitors: a retrospective study. J Cancer Res Clin Oncol.

[35] Wang DY, Salem J, Cohen JV, Chandra S, Menzer C, Ye F, Zhao S, Das S, Beckermann KE, Ha L, Rathmell WK, Ancell KK, Balko JM, Bowman C, Davis EJ, Chism DD, Horn L, Long GV, Carlino MS, Lebrun-Vignes B, Eroglu Z, Hassel JC, Menzies AM, Sosman JA, Sullivan RJ, Moslehi JJ, Johnson DB (2018). Fatal Toxic Effects Associated With Immune Checkpoint Inhibitors: A Systematic Review and Meta-analysis. JAMA Oncol.

[36] Basch E, Dueck AC, Rogak LJ, Minasian LM, Kelly WK, O'Mara AM, Denicoff AM, Seisler D, Atherton PJ, Paskett E, Carey L, Dickler M, Heist RS, Himelstein A, Rugo HS, Sikov WM, Socinski MA, Venook AP, Weckstein DJ, Lake DE, Biggs DD, Freedman RA, Kuzma C, Kirshner JJ, Schrag D (2017). Feasibility Assessment of Patient Reporting of Symptomatic Adverse Events in Multicenter Cancer Clinical Trials. JAMA Oncol.

[37] Basch E, Barbera L, Kerrigan CL, Velikova G (2018). Implementation of Patient-Reported Outcomes in Routine Medical Care. Am Soc Clin Oncol Educ Book.

[38] Basch E, Rogak LJ, Dueck AC (2016). Methods for Implementing and Reporting Patient-reported Outcome (PRO) Measures of Symptomatic Adverse Events in Cancer Clinical Trials. Clin Ther.

[39] Basch E, Dueck AC, Rogak LJ, Mitchell SA, Minasian LM, Denicoff AM, Wind JK, Shaw MC, Heon N, Shi Q, Ginos B, Nelson GD, Meyers JP, Chang GJ, Mamon HJ, Weiser MR, Kolevska T, Reeve BB, Bruner DW, Schrag D (2018). Feasibility of Implementing the Patient-Reported Outcomes Version of the Common Terminology Criteria for Adverse Events in a Multicenter Trial: NCCTG N1048. JCO.

[40] Wright E, Selby P, Crawford M, Gillibrand A, Johnston C, Perren T, Rush R, Smith A, Velikova G, Watson K, Gould A, Cull A (2003). Feasibility and Compliance of Automated Measurement of Quality of Life in Oncology Practice. JCO.

[41] Velikova G, Wright EP, Smith AB, Cull A, Gould A, Forman D, Perren T, Stead M, Brown J, Selby PJ (1999). Automated Collection of Quality-of-Life Data: A Comparison of Paper and Computer Touch-Screen Questionnaires. JCO.

[42] Basch E, Artz D, Dulko D, Scher K, Sabbatini P, Hensley M, Mitra N, Speakman J, McCabe M, Schrag D (2005). Patient Online Self-Reporting of Toxicity Symptoms During Chemotherapy. JCO.

[43] Freeman-Keller M, Kim Y, Cronin H, Richards A, Gibney G, Weber JS (2015). Nivolumab in Resected and Unresectable Metastatic Melanoma: Characteristics of Immune-Related Adverse Events and Association with Outcomes. Clinical Cancer Research.

[44] Sanlorenzo M, Vujic I, Daud A, Algazi A, Gubens M, Luna SA, Lin K, Quaglino P, Rappersberger K, Ortiz-Urda S (2015). Pembrolizumab Cutaneous Adverse Events and Their Association With Disease Progression. JAMA Dermatol.

[45] Berner F, Bomze D, Diem S, Ali OH, Fässler M, Ring S, Niederer R, Ackermann CJ, Baumgaertner P, Pikor N, Cruz CG, van de Veen W, Akdis M, Nikolaev S, Läubli H, Zippelius A, Hartmann F, Cheng H, Hönger G, Recher M, Goldman J, Cozzio A, Früh M, Neefjes J, Driessen C, Ludewig B, Hegazy AN, Jochum W, Speiser DE, Flatz L (2019). Association of Checkpoint Inhibitor-Induced Toxic Effects With Shared Cancer and Tissue Antigens in Non-Small Cell Lung Cancer. JAMA Oncol.

[46] Lizée T, Basch E, Trémolières P, Voog E, Domont J, Peyraga G, Urban T, Bennouna J, Septans A, Balavoine M, Detournay B, Denis F (2019). Cost-Effectiveness of Web-Based Patient-Reported Outcome Surveillance in Patients With Lung Cancer. J Thorac Oncol.

